# Single-shot measurement of the orbital-angular-momentum spectrum of light

**DOI:** 10.1038/s41467-017-01215-x

**Published:** 2017-10-20

**Authors:** Girish Kulkarni, Rishabh Sahu, Omar S. Magaña-Loaiza, Robert W. Boyd, Anand K. Jha

**Affiliations:** 1 Department of Physics, Indian Institute of Technology, Kanpur, 208016 India; 2 The Institute of Optics, University of Rochester, Rochester, NY 14627 USA; 3 Department of Physics, University of Ottawa, Ottawa, ON Canada K1N6N5

## Abstract

The existing methods for measuring the orbital-angular-momentum (OAM) spectrum suffer from issues such as poor efficiency, strict interferometric stability requirements, and too much loss. Furthermore, most techniques inevitably discard part of the field and measure only a post-selected portion of the true spectrum. Here, we propose and demonstrate an interferometric technique for measuring the true OAM spectrum of optical fields in a single-shot manner. Our technique directly encodes the OAM-spectrum information in the azimuthal intensity profile of the output interferogram. In the absence of noise, the spectrum can be fully decoded using a single acquisition of the output interferogram, and, in the presence of noise, acquisition of two suitable interferograms is sufficient for the purpose. As an important application of our technique, we demonstrate measurements of the angular Schmidt spectrum of the entangled photons produced by parametric down-conversion and report a broad spectrum with the angular Schmidt number 82.1.

## Abstract (web summary)

Efficient measurement of orbital angular momentum spectra presents open challenges. Here, the authors demonstrate a highly-efficient interferometric technique in which the OAM-spectrum information is encoded in the azimuthal intensity profile of the output interferogram.

## Introduction

It was shown by Allen et al. that a photon in a light beam can have orbital-angular-momentum (OAM) values in the integer multiples of *ħ* ^1^. This result has made OAM a very important degree of freedom for both classical and quantum information protocols^2–11^. This is because an information protocol requires a discrete basis, and the OAM degree of freedom provides a basis that is not only discrete but can also be high-dimensional^12–15^. This is in contrast to the polarization degree of freedom, which provides a discrete but only a two-dimensional basis^2–4^. High-dimensional quantum information protocols have many distinct advantages in terms of security^16–18^, transmission bandwidth^19, 20^, gate implementations^21, 22^, supersensitive measurements^23^, and fundamental tests of quantum mechanics^24–27^. In the classical domain, the high-dimensional OAM-states can increase the system capacities and spectral efficiencies^9–11^.

One of the major challenges faced in the implementation of OAM-based high-dimensional protocols is the efficient detection of OAM spectrum, and it is currently an active research area^28–37^. There are several approaches to measuring the OAM spectrum of a field. One main approach^28, 29^ is to display a specific hologram onto a spatial light modulator (SLM) for a given input OAM-mode and then measure the intensity at the first diffraction order using a single-mode fiber. This way, by placing different holograms specific to different input OAM-modes in a sequential manner, one is able to measure the spectrum. However, this method is very inefficient since the required number of measurements scales with the size of the input spectrum. Moreover, due to the non-uniform fiber-coupling efficiencies of different input OAM-modes^38^, this method does not measure the true OAM spectrum. The second approach relies on measuring the angular coherence function of the field and then reconstructing the OAM-spectrum through an inverse Fourier transform. One way to measure the angular coherence function is by measuring the interference visibility in a Mach–Zehnder interferometer as a function of the Dove-prism rotation angle^31, 32^. Although this method does not have any coupling-efficiency issue, it still requires a series of measurements for obtaining the angular coherence function. This necessarily requires that the interferometer be kept aligned for the entire range of the rotation angles. A way to bypass the interferometric stability requirement is by measuring the angular coherence function^33, 34^ using angular double-slits^39^. However, this method also requires a series of measurements and since in this method only a very small portion of the incident field is used for detection, it is not suitable for very low-intensity fields such as the fields produced by parametric down-conversion (PDC). The other approaches to measuring the OAM spectrum include techniques based on rotational Doppler frequency shift^35, 36^ and concatenated Mach–Zehnder interferometers^37^. However, due to several experimental challenges, these approaches^35–37^ have so far been demonstrated only for fields consisting of just a few modes. Thus the existing methods for measuring the OAM spectrum information suffer from either poor efficiency^28, 35^ or strict interferometric stability requirements^31, 32, 37^ or too much loss^33, 34^. In addition, many techniques^28, 33, 34^ inevitably discard part of the field and yield only a post-selected portion of the true spectrum.

In this article, we demonstrate an interferometric technique for measuring the true OAM spectrum in a single-shot manner, that is, by acquiring only one image of the output interferogram using a multi-pixel camera. Since our method is interferometric, the efficiency is very high, and since it involves only single-shot measurements, the interferometric stability requirements are much less stringent.

## Results

### Theory of single-shot spectrum measurement

The Laguerre-Gaussian (LG) modes, represented as ${\rm{LG}}_p^l\left( {\rho ,\phi } \right)$, are exact solutions of the paraxial Helmholtz equation. The OAM-mode index *l* measures the OAM of each photon in the units of *ħ*, while the index *p* characterizes the radial variation in the intensity^1^. The partially coherent fields that we consider in this article are the ones that can be represented as incoherent mixtures of LG modes having different OAM-mode indices^33^. The electric field *E*_in_(*ρ*, *ϕ*) corresponding to such a field can be written as

1 $${E_{{\rm{in}}}}\left( {\rho ,\phi } \right) = \mathop {\sum}\limits_{l,p} {A_{lp}}{\rm{LG}}_p^l\left( {\rho ,\phi } \right) = \mathop {\sum}\limits_{l,p} {A_{lp}}{\rm{LG}}_p^l(\rho ){e^{il\phi }},$$

where *A*_*lp*_ are stochastic variables. The corresponding correlation function *W*(*ρ*_1_, *ϕ*_1_; *ρ*_2_, *ϕ*_2_) is

2 $$\begin{array}{*{20}{l}} {W\left( {{\rho _1},{\phi _1};{\rho _2},{\phi _2}} \right)} &\equiv {{{\left\langle {E_{{\rm{in}}}^{\rm{*}}\left( {{\rho _1},{\phi _1}} \right){E_{{\rm{in}}}}\left( {{\rho _2},{\phi _2}} \right)} \right\rangle }_e}} \hskip3pc\\ &= {\mathop {\sum}\limits_{l,p,p'} {\alpha _{lpp'}}LG_p^{*l}\left( {{\rho _1},{\phi _1}} \right)LG_{p'}^l\left( {{\rho _2},{\phi _2}} \right).} \end{array}$$

Here ${\left\langle \cdots \right\rangle _e}$ represents the ensemble average and ${\langle {A_{lp}^*{A_{l'p'}}} \rangle _e} = {\alpha _{lpp'}}{\delta _{l,l'}}$, where *δ*_*l,l*′_ is the Kronecker-delta function. When integrated over the radial coordinate, the above correlation function yields the angular coherence function: $W\left( {{\phi _1},{\phi _2}} \right) \equiv {\int}_0^\infty \rho d\rho W\left( {\rho ,{\phi _1};\rho ,{\phi _2}} \right)$, which, for the above field, can be shown to be^33^

3 $$W\left( {{\phi _1},{\phi _2}} \right) \to W(\Delta \phi ) = \frac{1}{{2\pi }}\mathop {\sum}\limits_{l = - \infty }^\infty {S_l}{e^{ - il\Delta \phi }},$$

where ${S_l} = \mathop {\sum}\nolimits_p {\alpha _{lpp}}$, Δ*ϕ* = *ϕ*_1_ − *ϕ*_2_, and where we have used the identity ${\int}_{0}^\infty \rho {\rm{LG}}_p^{*l}\left( \rho \right){\rm{LG}}_{p'}^l\left( \rho \right){d}\rho = {\delta _{pp'}}{\rm{/}}2\pi$. The quantity *S*_*l*_ is referred to as the OAM spectrum of the field. It is normalized such that $\mathop {\sum}\nolimits_l {S_l} = 1$ and ${\int}_{ - \pi }^\pi W\left( {{\phi _1},{\phi _1}} \right)d{\phi _1} = 1$. The Fourier transform relation of Eq. (3) is the angular analog of the temporal Wiener–Khintchine theorem for temporally stationary fields (see Section 2.4 of ref. ^40^). Therefore, a measurement of *W(*Δ*ϕ*) can yield the OAM spectrum of the input field through the inverse Fourier relation

4 $${S_l} = {\int}_{\!\!\!\! - \pi }^\pi W\left( {\Delta \phi } \right){e^{il\Delta \phi }}d\Delta \phi .$$

**Fig. 1:**
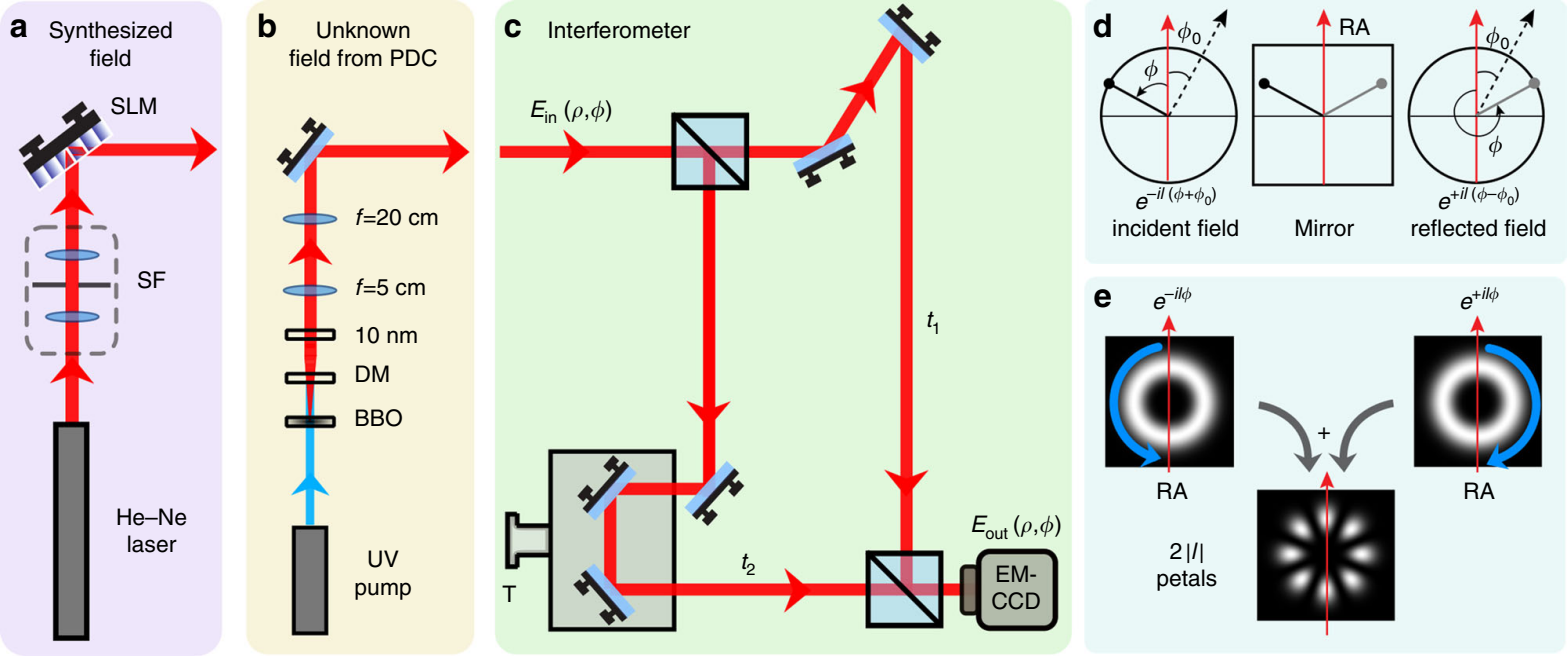
Describing the proposed experimental technique and its working priciple. **a** Schematic of the setup for synthesizing partially coherent fields of the type represented by Eqs. (1) and (2) with known OAM spectra. **b** Schematic of the setup for producing the partially coherent fields through parametric down-conversion. **c** Schematic of the Mach–Zehnder interferometer. **d** Describing how a mirror reflection changes the azimuthal phase of an OAM mode. An incident beam with the azimuthal phase profile ${e^{il\left( {\phi + {\phi _0}} \right)}}$ transforms into a beam having the azimuthal phase profile ${e^{ - il\left( {\phi - {\phi _0}} \right)}}$, where *ϕ*_0_ is the angle between the reflection axis (RA) and the zero-phase axis (dashed axis) of the incident mode. **e** Illustrating the interference effect produced by the interferometer when the incident field is an $LG_{p = 0}^l(\rho ,\phi )$ mode with *l* = 4. At the output, we effectively have the interference of an *e* ^*ilϕ*^ mode with an *e* ^−*ilϕ*^ mode, and we obtain the output interference intensity in the form of a petal pattern with the number of petals being 2$\left| l \right|$ = 8. SLM: spatial light modulator; SF: spatial filter; DM: dichroic mirror; BBO: type-I beta barium borate crystal; T: translation stage

Now, let us consider the situation shown in Fig. 1a. A partially coherent field of the type represented by Eqs. (1) and (2) enters the Mach–Zehnder interferometer having an odd and an even number of mirrors in the two arms (shown in Fig. 1c). As illustrated in Fig. 1d, each reflection transforms the polar coordinate as *ρ*→*ρ* and the azimuthal coordinate as *ϕ* + *ϕ*_0_→−*ϕ* + *ϕ*_0_ across the reflection axis (RA). Here *ϕ* is the angle measured from RA, and *ϕ*_0_ is the angular-separation between RA and the zero-phase axis of the incident mode (dashed axis). The phase *ϕ*_0_ does not survive in intensity expressions. So, without the loss of any generality, we take *ϕ*_0_ = 0 for all incident modes. Therefore, for the input incident field *E*_in_(*ρ*, *ϕ*) of Eq. (1), the field *E*_out_(*ρ*, *ϕ*) at the output port becomes

5 $$\begin{array}{*{20}{l}} {{E_{{\rm{out}}}}\left( {\rho ,\phi } \right)}\!\!\!\!\! & = {\sqrt {{k_1}} {E_{{\rm{in}}}}\left( {\rho , - \phi } \right){e^{i\left( {{\omega _0}{t_1} + {\beta _1}} \right)}}} \\ & { + \sqrt {{k_2}} {E_{{\rm{in}}}}\left( {\rho ,\phi } \right){e^{i\left( {{\omega _0}{t_2} + {\beta _2} + \tilde \gamma } \right)}}.} \hfill \\ \end{array}$$

Here, *t*_1_ and *t*_2_ denote the travel-times in the two arms of the interferometer; *ω*_0_ is the central frequency of the field; *β*_1_ and *β*_2_ are the phases other than the dynamical phase acquired in the two arms; $\tilde \gamma$ is a stochastic phase which incorporates the temporal coherence between the two arms; *k*_1_ and *k*_2_ are the scaling constants in the two arms, which depend on the splitting ratios of the beam splitters, etc. The azimuthal intensity *I*_out_ *(ϕ*) at the output port is defined as ${I_{{\rm{out}}}}(\phi ) \equiv {\int} \rho {\left\langle {E_{{\rm{out}}}^{\rm{*}}\left( {\rho ,\phi } \right)E_{{\rm{out}}}^{\rm{*}}\left( {\rho ,\phi } \right)} \right\rangle _e}{d}\rho$, and using Eqs. (1)–(4), we can evaluate it to be

6 $${I_{{\rm{out}}}}(\phi ) = \frac{1}{{2\pi }}\left( {{k_1} + {k_2}} \right) + \gamma \sqrt {{k_1}{k_2}} W(2\phi ){e^{i\delta }} + {\rm{c}}.{\rm{c}}{\rm{.}}$$

Here, we have defined *δ* ≡ *ω*_0_(*t*_2_ − *t*_1_) + (*β*_2_ − *β*_1_), and $\gamma = \left\langle {{e^{i\tilde \gamma }}} \right\rangle$ quantifies the degree of temporal coherence. The intensity expression in Eq. (6) is very different from the output intensity expression one obtains in a conventional Mach–Zehnder interferometer with a Dove prism having either odd/odd or even/even number of mirrors in the two interferometric arms^31, 32^. In Eq. (6), the output intensity and the angular correlation function both depend on the detection-plane azimuthal angle *ϕ*. As a result, the angular correlation function *W*(2*ϕ*) comes out encoded in the azimuthal intensity profile *I*_out_ *(ϕ*). In contrast, in the conventional Mach–Zehnder interferometers^31, 32^, the output intensity has no azimuthal variation; one measures the angular correlation function by measuring the interference visibility of the total output intensity as a function of the Dove prism rotation angles. For a symmetric spectrum (*S*_*l*_ = *S*_−*l*_ = (*S*_*l*_ + *S*_−*l*_)/2), we have, using the formula in Eq. (4)

7 $$\begin{array}{*{20}{l}} {{S_l}} = {{\int}_{\! - \pi }^\pi W(2\phi ){e^{i2l\phi }}{d}(2\phi )} \\ = {\frac{1}{{2\gamma \,{\rm{cos}}\,\delta \sqrt {{k_1}{k_2}} }}{\int}_{\! - \pi }^\pi \left[ {{I_{{\rm{out}}}}(\phi ) - \frac{{{k_1} + {k_2}}}{{2\pi }}} \right]{\rm{cos}}(2l\phi ){d}\phi .} \hfill \\ \end{array}$$

So, if the precise values of *k*_1_, *k*_2_, *γ*, and *δ* are known then a single-shot measurement of the output interferogram *I*_out_(*ϕ*) yields the angular coherence function *W*(2*ϕ*) and thereby the OAM spectrum *S*_*l*_. Here, by “a single-shot measurement” we mean recording one image of the output interferogram using a multi-pixel camera. The recording may involve collecting several photons per pixel for a fixed exposure time of the camera.

### Theory of two-shot noise-insensitive spectrum measurement

Although it is in principle possible to measure the OAM spectrum in a single-shot manner as discussed above, it is practically extremely difficult to do so because of the requirement of a very precise knowledge of *k*_1_, *k*_2_, *γ*, and *δ*. Moreover, obtaining a spectrum in this manner is susceptible to noise in the measured *I*_out_(*ϕ*), which results in errors in the measured spectrum. We now show that it is possible to eliminate this noise completely while also relinquishing the need for a precise knowledge of *k*_1_, *k*_2_, *γ*, and *δ*, just by acquiring one additional output interferogram. We present our analysis for a symmetric spectrum, that is, for (*S*_*l*_ = *S*_−*l*_ = (*S*_*l*_ + *S*_−*l*_)/2) (see Methods section for the non-symmetric case). Let us assume that the experimentally measured output azimuthal intensity $\bar I_{{\rm{out}}}^\delta (\phi )$ at *δ* contains some noise $I_{\rm{n}}^\delta (\phi )$ in addition to the signal *I*_out_(*ϕ*), that is,

$$\bar I_{{\rm{out}}}^\delta (\phi ) = I_{\rm{n}}^\delta (\phi ) + \frac{1}{{2\pi }}\left( {{k_1} + {k_2}} \right) + 2\gamma \sqrt {{k_1}{k_2}} W(2\phi ){\rm{cos}}\,\delta .$$

Now, suppose that we have two interferograms, $\bar I_{{\rm{out}}}^{{\delta _c}}(\phi )$ and $\bar I_{{\rm{out}}}^{{\delta _d}}(\phi )$, measured at *δ* *=* *δ*_*c*_ and *δ* *=* *δ*_*d*_, respectively. The difference in the intensities $\Delta {\bar I_{{\rm{out}}}}(\phi ) = \bar I_{{\rm{out}}}^{{\delta _c}}(\phi ) - \bar I_{{\rm{out}}}^{{\delta _d}}(\phi )$ of the two interferograms is then given by

$$\Delta {\bar I_{{\rm{out}}}}(\phi ) = \Delta {I_{\rm{n}}}(\phi ) + 2\gamma \sqrt {{k_1}{k_2}} \left( {{\rm{cos}}\,{\delta _c} - {\rm{cos}}\,{\delta _d}} \right)W(2\phi ),$$

where $\Delta {I_{\rm{n}}}(\phi ) = I_{\rm{n}}^{{\delta _c}}(\phi ) - I_{\rm{n}}^{{\delta _d}}(\phi )$ is the difference in the noise intensities. Multiplying each side of the above equation by *e* ^*i*2*lϕ*^, using the formula in Eq. (4), and defining the measured OAM spectrum as ${\bar S_l} \equiv {\int}_{ - \pi }^\pi \Delta {\bar I_{{\rm{out}}}}(\phi ){e^{i2l\phi }}d(2\phi ) = {\int}_{ - \pi }^\pi \Delta {\bar I_{{\rm{out}}}}(\phi ){e^{i2l\phi }}d\phi$, we get

8 $${\bar S_l} = {\int}_{\!\!\!\!\! - \pi }^\pi \Delta {I_{\rm{n}}}(\phi ){e^{i2l\phi }}d\phi + 2\gamma \sqrt {{k_1}{k_2}} \left( {{\rm{cos}}\,{\delta _c} - {\rm{cos}}{\delta _d}} \right){S_l}.$$

We see that in situations in which there is no shot-to-shot variation in noise, that is, Δ*I*_n_(*ϕ*) = 0, the measured OAM-spectrum ${\bar S_l}$ is same as the true input OAM-spectrum *S*_*l*_ up to a scaling constant. One can thus obtain the normalized OAM-spectrum in a two-shot manner without having to know the exact values of *k*_1_, *k*_2_, *γ*, *δ*_*c*_, or *δ*_*d*_. Nevertheless, in order to get a better experimental signal-to-noise ratio, it would be desirable to have *γ* ≈ 1, *k*_1_ ≈ *k*_2_ ≈ 0.5, *δ*_*c*_ ≈ 0, and *δ*_*d*_ ≈ *π*. Now, in situation in which Δ*I*_n_(*ϕ*) ≠ 0, it is clear from Eq. (8) that the measured spectrum will have extra contributions. However, since we do not expect very rapid azimuthal variations in Δ*I*_n_(*ϕ*), the extra contributions should be more prominent for modes around *l* = 0 and should die down for large-*l* modes.

### Measuring lab-synthesized OAM spectra

**Fig. 2:**
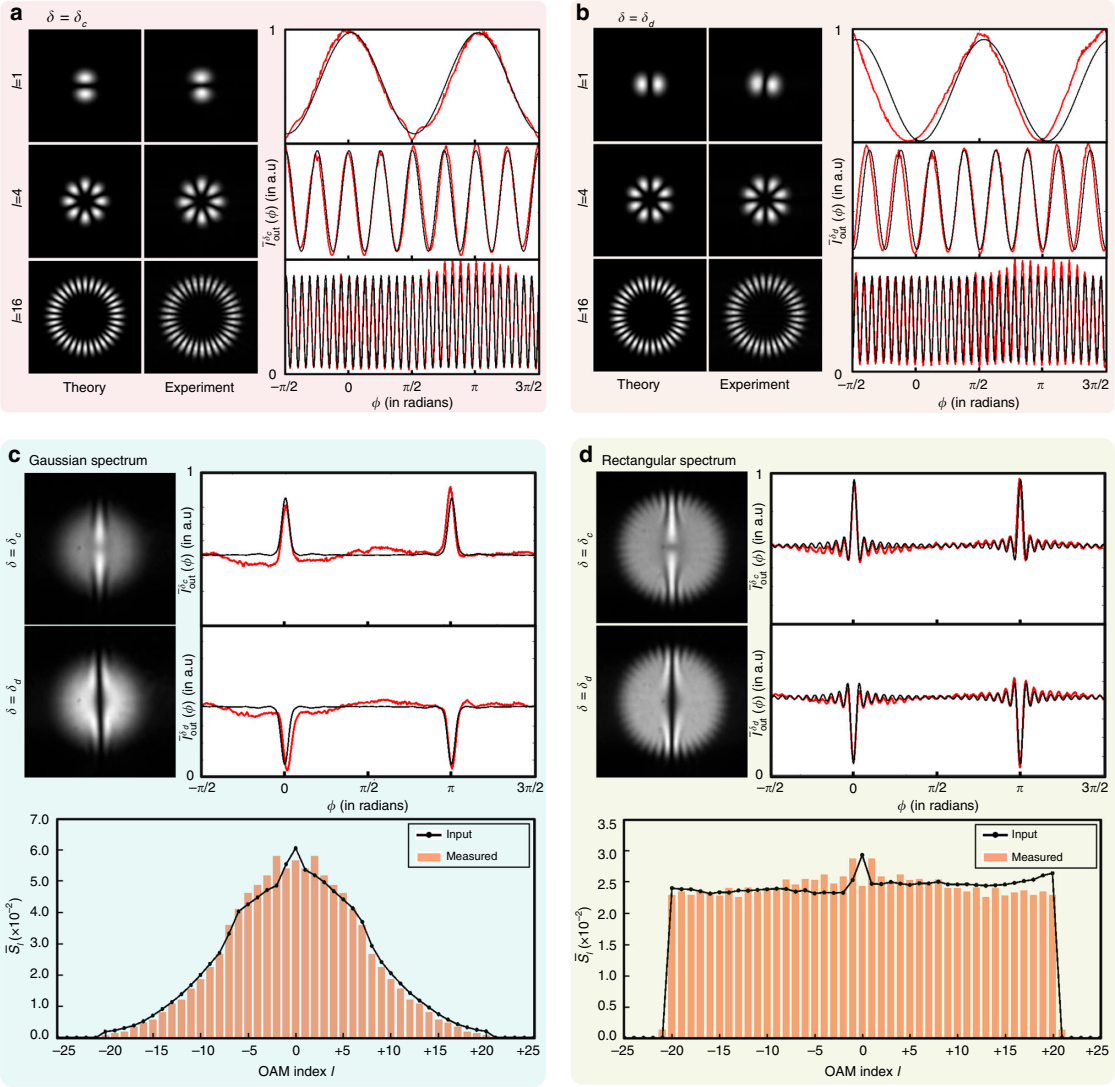
Experimental results obtained with the lab-synthesized fields. **a**, **b** Measured output interferograms and the corresponding azimuthal intensities for input $LG_{p = 0}^l\left( {\rho ,\phi } \right)$ modes with *l* = 1, 4, and 16 for *δ*_*c*_ ≈ 2*mπ* and *δ*_*d*_ ≈ (2*m* + 1)*π*, respectively, where *m* is an integer. **c** Measured output interferograms, the azimuthal intensities, and the measured spectrum for the synthesized input field with a Gaussian OAM-spectrum. **d** Measured output interferograms, the azimuthal intensities, and the measured spectrum for the synthesized input field with a Rectangular OAM-spectrum. In all the above azimuthal intensity plots, the red lines are the experimental plots and the black lines are the theoretical fits

We now report the experimental demonstrations of our technique for laboratory-synthesized, symmetric OAM spectra. As shown in Fig. 1a, a He–Ne laser is spatially filtered and made incident onto a Holoeye Pluto SLM. The ${\rm{LG}}_{p = 0}^l(\rho ,\phi )$ modes are generated using the method by Arrizon et al.^41^ and then made incident into the Mach–Zehnder interferometer shown in Fig. 1c. The measured interferogram and the corresponding azimuthal intensity $\bar I_{{\rm{out}}}^\delta (\phi )$ for a few ${\rm{LG}}_{p = 0}^l(\rho ,\phi )$ modes for *δ*_*c*_ ≈ 2*mπ*, and *δ*_*d*_ ≈ (2*m* + 1)*π*, where *m* is an integer, are presented in Fig. 2a, b, respectively. A very good match between the theory and experiment indicates that the ${\rm{LG}}_{p = 0}^l(\rho ,\phi )$ modes produced in our experiments are of very high quality. By controlling the strengths of the synthesized ${\rm{LG}}_{p = 0}^l(\rho ,\phi )$ modes for *l* ranging from *l* = −20 to *l* = 20, we synthesize two separate fields: one with a rectangular spectrum and the other one with a Gaussian spectrum. The representative interferogram corresponding to a particular field as input is obtained by adding individual interferograms for *l* ranging from *l* = −20 to *l* = +20. Two such representative interferograms, one with *δ* = *δ*_*c*_ and other one with *δ* = *δ*_*d*_ are recorded for each field. Figure 2c, d show the measured output interferograms, the corresponding azimuthal intensities, and the measured spectrum ${\bar S_l}$ computed using Eq. (8) for the synthesized Gaussian and Rectangular OAM spectra, respectively. We find a very good match between the synthesized spectra and the measured spectra. There is some mismatch in the measured spectra for low-*l* modes. We attribute this to SLM imperfections, various wave-front aberrations, and the non-zero shot-to-shot noise variation Δ*I*_n_(*ϕ*).

### Measuring angular Schmidt spectrum of entangled states

The state of the two-photon field produced by PDC has the following Schmidt-decomposed form when the detection system is sensitive only to the OAM-mode index^33^:

9 $$\left| { {\psi _2}} \right\rangle = \mathop {\sum}\limits_{l = - \infty }^\infty \sqrt {{S_l}} {\left| l \right\rangle _s}{\left| { - l} \right\rangle _i}.$$

Here *s* and *i* stand for signal and idler photons, respectively, $\left| l \right\rangle$ represents a mode with OAM-mode index *l*, and *S*_*l*_ is referred to as the angular Schmidt spectrum. The angular Schmidt spectrum quantifies the dimensionality and the entanglement of the state in the OAM basis^32, 42, 43^. There are a variety of techniques for measuring the angular Schmidt spectrum^28, 32, 33, 44, 45^. In the context of spatial entanglement, there has even been a theoretical proposal^46^ and its subsequent experimental implementation^47^ for measuring the spatial Schmidt spectrum in a single-shot manner using coincidence detection. However, all the above mentioned work, including the single-shot work in the spatial domain^46, 47^, are sensitive to noise and require either a very precise knowledge of the experimental parameters, such as beam splitting ratio, or a very stable interferometer. In contrast, as an important experimental application of our technique, we now report an experimental measurement of the angular Schmidt spectrum of the PDC photons that not only is a single-shot, noise-insensitive technique but also does not require coincidence detection.

**Fig. 3:**
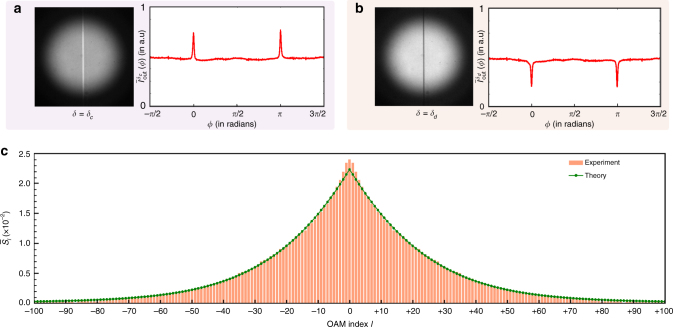
Experimental results obtained with fields produced by parametric down-conversion. **a**, **b** Measured output interferogram and the azimuthal intensity for *δ* = *δ*_*c*_ and *δ* = *δ*_*d*_, respectively. **c** The normalized measured spectrum ${\bar S_l}$ as computed using Eq. (8), and the normalized theoretical spectrum as calculated using the formalism of ref. ^42^, for our setup parameters, namely, a type-I collinear down-conversion with a 2-mm thick BBO crystal and a 0.85-mm beam-waist pump laser. The theoretical spectrum has no fitting parameters. The angular Schmidt number $K = 1{\rm{/}}\left( {\mathop {\sum}\nolimits_l \bar S_l^2} \right)$ for the measured spectrum is evaluated to be 82.1

As derived in ref. ^33^, the angular coherence function *W*_*s*_(*ϕ*_1_, *ϕ*_2_) corresponding to the individual signal or idler photon has the following form:

10 $${W_s}\left( {{\phi _1},{\phi _2}} \right) \to {W_s}(\Delta \phi ) = \frac{1}{{2\pi }}\mathop {\sum}\limits_{l = - \infty }^\infty {S_l}{e^{ - il\Delta \phi }},$$

where ${S_l} = \mathop {\sum}\nolimits_p {\alpha _{lpp}}$ is the OAM spectrum of individual photons. Comparing Eqs. (9) and (10), we find that the OAM spectrum of individual photons is same as the angular Schmidt spectrum of the entangled state. Therefore, it is clear that one can measure the angular Schmidt spectrum in a single-shot manner by measuring the OAM-spectrum of individual photons in a single-shot manner. As depicted in Fig. 1b, entangled photons are produced by PDC with collinear type-I phase matching. The photons are collected by a lens arrangement whose collection angle is larger than the emission cone-angle of the crystal. This ensures that no part of the produced field is discarded from the measurement and thus that the true spectrum is measured. This field is then made incident into the Mach–Zehnder interferometer of Fig. 1b. For a type-I collinear down-conversion with a 2-mm thick beta barium borate (BBO) crystal and a 0.85-mm beam-waist pump laser, the measured output interferograms and the corresponding azimuthal intensities for two values of *δ* have been shown in Fig. 3a, b. Figure 3c shows the normalized measured spectrum as computed using Eq. (8) and the normalized theoretical spectrum as calculated using the formalism of ref. ^42^. The near-perfect match without the use of any fitting parameter shows that we have indeed measured the true theoretical angular Schmidt spectrum of PDC photons. There is some mismatch for low-*l* values, which we attribute to the very small but finite shot-to-shot noise variation Δ*I*_n_(*ϕ*). The angular Schmidt number computed as $K{\kern 1pt} = {\kern 1pt} 1{\rm{/}}\left( {\mathop {\sum}\nolimits_l \bar S_l^2} \right)$ is *K* = 82.1, which, to the best of our knowledge, is the highest-ever reported angular Schmidt number so far.

## Discussion

In conclusion, we have proposed and demonstrated a single-shot technique for measuring the angular coherence function and thereby the OAM spectrum of light fields that can be represented as mixtures of modes with different OAM values per photon. Our technique involves a Mach–Zehnder interferometer with the central feature of having an odd and an even number of mirrors in the two interferometric arms, and it is very robust to noise and does not require precise characterization of setup parameters, such as beam splitting ratios, degree of temporal coherence, etc. This technique also does not involve any inherent post-selection of the field to be measured and thus measures the true OAM spectrum of a field. As an important application of this technique, we have reported the measurement of a very high-dimensional OAM-entangled states in a single shot manner, without requiring coincidence detection. For such high-dimensional states, our technique improves the time required for measuring the OAM spectrum by orders of magnitude. This can have important implications in terms of improving the signal-to-noise ratio and reducing the interferometric stability requirements for both classical and quantum communication protocols that are based on using OAM of photons.

The technique presented in this article is for fields that can be represented as incoherent mixtures of modes carrying different OAM-mode indices. However, in many important applications, such as OAM-based multiplexing in communication protocols^9–11^, one uses fields that are coherent superpositions of OAM-carrying modes. For such fields, we believe that the generalizations of the reconstruction techniques^48, 49^ used for complex-valued objects could be a possible way of getting the state information in a single-shot manner. Moreover, in recent years, finding efficient ways for measuring a partially coherent field is becoming an important research pursuit^50^, and we believe that, at least in the OAM degree of freedom, the generalized versions of the existing techniques for coherent fields in combination with our technique presented in this article might pave the way towards a full quantum state tomography in a single-shot or a few-shots manner.

## Methods

### Details of the experiment with lab-synthesized fields

In this experiment, the ${\rm{LG}}_{p = 0}^l(\rho ,\phi )$ modes were generated by an SLM using the method by Arrizon et al^41^. These modes were made sequentially incident into the interferometer and the corresponding output interferograms were imaged using an Andor iXon Ultra electron-multiplied charge-coupled device (EMCCD) camera having 512 × 512 pixels. For each individual ${\rm{LG}}_{p = 0}^l(\rho ,\phi )$ mode the camera was exposed for about 0.4 s. The sequential acquisition was automated to ensure that *δ* is the same for all the modes. The azimuthal intensity $\bar I_{{\rm{out}}}^\delta (\phi )$ plots were obtained by first precisely positioning a very narrow angular region-of-interest (ROI) at angle *ϕ* in the interferogram image and then integrating the intensity within the ROI up to a radius that is sufficiently large. To reduce pixelation-related noise, the interferograms were scaled up in size by a factor of four using a bicubic interpolation method. In order to ensure minimal shot-to-shot noise variation, the interferometer was covered after the required alignment with a box and the measurements were performed only after it had stabilized in terms of ambient fluctuations.

### Details of the experiment measuring angular Schmidt spectrum

As depicted in Fig. 1b, a 405 nm ultraviolet pump laser with a beam radius 0.85 mm and having a Gaussian transverse mode profile was made incident onto a 2-mm thick BBO crystal. The crystal was phase-matched for collinear type-I PDC. The pump power of 100 mW ensured that we were working within the weak down-conversion limit, in which the probability of producing a four-photon state is negligibly small compared to that of producing a two-photon state. The residual pump photons after the crystal were discarded by means of a dichroic mirror. The down-converted photons were passed through an interference filter of spectral width 10 nm centered at 810 nm and then made incident into the Mach–Zehnder interferometer of Fig. 1b. The output of the interferometer was recorded using an Andor iXon Ultra EMCCD camera having 512 × 512 pixels with the acquisition time of 13 s. The azimuthal intensity $\bar I_{{\rm{out}}}^\delta (\phi )$ plots were obtained by first precisely positioning a very narrow angular ROI at angle *ϕ* in the interferogram image and then integrating the intensity within the ROI up to a radius that is sufficiently large. To reduce pixelation-related noise, the interferograms are scaled up in size by a factor of eight using a bicubic interpolation method.

We note that since we are using collinear down-conversion, the individual signal and idler photons have equal probability of arriving at a given output port of the interferometer. As a result, what is recorded by the camera at a given output port is the sum of the interferograms produced by the signal and idler fields at that output port. However, since the individual signal and idler fields have the same OAM spectrum, the azimuthal profile of the sum interferogram is same as that of the individual interferograms produced by either the signal or the idler field. One assumption that we have made here is that the probability of simultaneous arrivals of the signal and idler photons at the same EMCCD-camera pixel is negligibly small. This assumption seems perfectly valid given that the EMCCD camera has 512 × 512 pixels, and as shown in Fig. 3, the output interferograms occupy more than half of the EMCCD-camera pixels.

### Theory of non-symmetric OAM-spectrum measurement

This section presents our analysis for a non-symmetric spectrum, that is, when *S*_*l*_ = *S*_−*l*_ condition is not necessarily met. Just as in the case of symmetric spectrum, let us assume that the measured azimuthal intensity $\bar I_{{\rm{out}}}^\delta (\phi )$ at the output contains the noise term $I_{\rm{n}}^\delta (\phi )$ in addition to the signal *I*_out_(*ϕ*). Thus,

11 $$\begin{array}{*{20}{l}} {\bar I_{{\rm{out}}}^\delta (\phi )} = {I_{\rm{n}}^\delta (\phi ) + {I_{{\rm{out}}}}(\phi )} \\ {I_{\rm{n}}^\delta (\phi ) + \frac{{{k_1} + {k_2}}}{{2\pi }} + \gamma \sqrt {{k_1}{k_2}} \left[ {W(2\phi ){e^{ - i\delta }} + {\rm{c}}{\rm{.c}}{\rm{.}}} \right].} \hfill \\ \end{array}$$

Now, suppose we have two interferograms measured at two different values of *δ*, say at *δ*_*c*_ and *δ*_*d*_. The difference $\Delta {\bar I_{{\rm{out}}}}(\phi )$ in the intensities of the two interferograms is then given by

12 $$\begin{array}{*{20}{l}} {\Delta {{\bar I}_{{\rm{out}}}}(\phi )} \hfill & = \hfill & {\bar I_{{\rm{out}}}^{{\delta _c}}(\phi ) - \bar I_{{\rm{out}}}^{{\delta _d}}(\phi )} \hfill \\ {} \hfill & = \hfill & {\Delta {I_{\rm{n}}}(\phi \,) + \gamma \sqrt {{k_1}{k_2}} \left[ {W(2\phi ){e^{ - i{\delta _c}}} + {W^*}(2\phi ){e^{i{\delta _c}}}} \right.} \hfill \\ {} \hfill & {} \hfill & {\left. { - W(2\phi ){e^{ - i{\delta _d}}} - {W^*}(2\phi ){e^{i{\delta _d}}}} \right],} \hfill \\ \end{array}$$

where $\Delta {I_{\rm{n}}}(\phi ) = I_{\rm{n}}^{{\delta _c}}(\phi ) - I_{\rm{n}}^{{\delta _d}}(\phi )$ is the difference in the noise intensities. Unlike in the case of symmetric spectrum, $\Delta {\bar I_{{\rm{out}}}}(\phi )$ is not proportional to the angular coherence function *W(*2*ϕ*). Multiplying each side of Eq. (12) by *e* ^*i*2*lϕ*^ and using the angular Wiener–Khintchine relation ${S_l} = {\int}_{\! - \pi }^\pi W(2\phi ){e^{i2l\phi }}d(2\phi )$, we obtain

13 $$\begin{array}{*{20}{l}} {{\int}_{\! - \pi }^\pi \Delta {{\bar I}_{{\rm{out}}}}(\phi ){e^{i2l\phi }}d(2\phi )} \hfill & = \hfill & {{\int}_{\! - \pi }^\pi \Delta {I_{\rm{n}}}(\phi ){e^{i2l\phi }}d(2\phi )} \hfill \\ {} \hfill & {} \hfill & { + \gamma \sqrt {{k_1}{k_2}} \left[ {{S_l}{e^{ - i{\delta _c}}} + {S_{ - l}}{e^{i{\delta _c}}} - {S_l}{e^{ - i{\delta _d}}} - {S_{ - l}}{e^{i{\delta _d}}}} \right].} \hfill \\ \end{array}$$

Now, multiplying each side of Eq. (12) by *e* ^−*i*2*lϕ*^ and using the angular Wiener–Khintchine relation ${S_l} = {\int}_{\! - \pi }^\pi W(2\phi ){e^{i2l\phi }}d(2\phi )$, we obtain

14 $$\begin{array}{*{20}{l}} {{\int}_{\! - \pi }^\pi \Delta {{\bar I}_{{\rm{out}}}}(\phi ){e^{ - i2l\phi }}d(2\phi )} \hfill & = \hfill & {{\int}_{\! - \pi }^\pi \Delta {I_{\rm{n}}}(\phi ){e^{ - i2l\phi }}d(2\phi )} \hfill \\ {} \hfill & {} \hfill & { + \gamma \sqrt {{k_1}{k_2}} \left[ {{S_{ - l}}{e^{ - i{\delta _c}}} + {S_l}{e^{i{\delta _c}}} - {S_{ - l}}{e^{ - i{\delta _d}}} - {S_l}{e^{i{\delta _d}}}} \right].} \hfill \\ \end{array}$$

Adding Eqs. (13) and (14), we get

15 $$\begin{array}{*{20}{l}} {{\int}_{\! - \pi }^\pi \Delta {{\bar I}_{{\rm{out}}}}(\phi ){\rm{cos}}(2l\phi )d(2\phi )} \hfill & = \hfill & {{\int}_{\! - \pi }^\pi \Delta {I_{\rm{n}}}(\phi ){\rm{cos}}(2l\phi )d(2\phi )} \hfill \\ {} \hfill & {} \hfill & { + \gamma \sqrt {{k_1}{k_2}} \left( {{S_l} + {S_{ - l}}} \right)\left( {{\rm{cos}}\,{\delta _c} - {\rm{cos}}\,{\delta _d}} \right).} \hfill \\ \end{array}$$

Subtracting Eq. (14) from Eq. (13), we get

16 $$\begin{array}{*{20}{l}} {{\int}_{\! - \pi }^\pi \Delta {{\bar I}_{{\rm{out}}}}(\phi ){\rm{sin}}(2l\phi )d(2\phi )} \hfill & = \hfill & {{\int}_{\! - \pi }^\pi \Delta {I_{\rm{n}}}(\phi ){\rm{sin}}(2l\phi )d(2\phi )} \hfill \\ {} \hfill & {} \hfill & { - \gamma \sqrt {{k_1}{k_2}} \left( {{S_l} - {S_{ - l}}} \right)\left( {{\rm{sin}}\,{\delta _c} - {\rm{sin}}\,{\delta _d}} \right).} \hfill \\ \end{array}$$

Now the question is how should one define the spectrum so that the defined spectrum becomes proportional to the true spectrum. Upon inspection we find that for the non-symmetric case it is not possible to define the spectrum the way we did it in the case of symmetric spectrum. Nevertheless, in the special situation in which *δ*_*c*_ + *δ*_*d*_ = *π*/2, it is possible to define the measured spectrum just like we did it in the symmetric case. Let us consider the situation when *δ*_*c*_ = *θ* and *δ*_*d*_ = *π*/2 − *θ* such that *δ*_*c*_ + *δ*_*d*_ = *π*/2. Equations (15) and (16) for this situation can be written as

17 $$\begin{array}{*{20}{l}} {{\int}_{\! - \pi }^\pi \Delta {{\bar I}_{{\rm{out}}}}(\phi ){\rm{cos}}(2l\phi )d(2\phi )} \hfill & = \hfill & {{\int}_{\! - \pi }^\pi \Delta {I_{\rm{n}}}(\phi ){\rm{cos}}(2l\phi )d(2\phi )} \hfill \\ {} \hfill & {} \hfill & { + \gamma \sqrt {{k_1}{k_2}} \left( {{S_l} + {S_{ - l}}} \right)({\rm{cos}}\,\theta - {\rm{sin}}\,\theta ).} \hfill \\ \end{array}$$

and

18 $$\begin{array}{*{20}{l}} {{\int}_{\! - \pi }^\pi \Delta {{\bar I}_{{\rm{out}}}}(\phi ){\rm{sin}}(2l\phi )d(2\phi )} \hfill & = \hfill & {{\int}_{\! - \pi }^\pi \Delta {I_{\rm{n}}}(\phi ){\rm{sin}}(2l\phi )d(2\phi )} \hfill \\ {} \hfill & {} \hfill & { + \gamma \sqrt {{k_1}{k_2}} \left( {{S_l} - {S_{ - l}}} \right)({\rm{cos}}\,\theta - {\rm{sin}}\,\theta ).} \hfill \\ \end{array}$$

Adding Eqs. (17) and (18), we get

19 $$\begin{array}{l} {\int}_{\!\! - \pi }^\pi \Delta {{\bar I}_{{\rm{out}}}}(\phi )\left[ {{\rm{cos}}(2l\phi ) + {\rm{sin}}(2l\phi )} \right]d(2\phi )\\ = {\int}_{\!\!\!\! - \pi }^\pi \Delta {I_{\rm{n}}}(\phi )\left[ {{\rm{cos}}(2l\phi ) + {\rm{sin}}(2l\phi )} \right]d(2\phi )\\ + 2\gamma \sqrt {{k_1}{k_2}} \left( {{\rm{cos}}\,\theta - {\rm{sin}}\,\theta } \right){S_l}.\\ \end{array}$$

So, now if we define the measured spectrum ${\bar S_l}$ to be

20 $$\begin{array}{*{20}{l}} {{{\bar S}_l}} \hfill & \equiv \hfill & {{\int}_{\!\! - \pi }^\pi \Delta {{\bar I}_{{\rm{out}}}}(\phi )\left[ {{\rm{cos}}(2l\phi ) + {\rm{sin}}(2l\phi )} \right]d(2\phi )} \hfill \\ {} \hfill & = \hfill & {{\int}_{\!\! - \pi /2}^{\pi /2} 2\Delta {{\bar I}_{{\rm{out}}}}(\phi )\left[ {{\rm{cos}}(2l\phi ) + {\rm{sin}}(2l\phi )} \right]d\phi } \hfill \\ {} \hfill & = \hfill & {{\int}_{\!\! - \pi }^\pi \Delta {{\bar I}_{{\rm{out}}}}(\phi )\left[ {{\rm{cos}}(2l\phi ) + {\rm{sin}}(2l\phi )} \right]d\phi ,} \hfill \\ \end{array}$$

we get,

21 $$\begin{array}{*{20}{l}} {{{\bar S}_l}} \hfill & = \hfill & {{\int}_{\!\! - \pi }^\pi \Delta {I_{\rm{n}}}(\phi )\left[ {{\rm{cos}}(2l\phi ) + {\rm{sin}}(2l\phi )} \right]d\phi } \hfill \\ {} \hfill & {} \hfill & { + 2\gamma \sqrt {{k_1}{k_2}} ({\rm{cos}}\,\theta - {\rm{sin}}\theta ){S_l}.} \hfill \\ \end{array}$$

In situations in which the noise neither has any explicit functional dependence on *δ* nor has any shot-to-shot variation, we have Δ*I*_n_(*ϕ*) = 0. Thus the defined spectrum ${\bar S_l}$ becomes proportional to the true spectrum *S*_*l*_. We see that just as in the case of symmetric spectrum, one does not have to know the exact values of *k*_1_, *k*_2_, *γ*, and *θ*. The only thing different in this case is that one has to take the two shots such that *δ*_*c*_ + *δ*_*d*_ = *π*/2.

### Data availability

The authors declare that the main data supporting the findings of this study are available within the article. Extra data are available from the corresponding author upon request.
